# Supplementary material for: Novel Long Non-Coding RNA (lncRNA) Transcript AL137782.1 Promotes the Migration of Normal Lung Epithelial Cells through Positively Regulating LMO7
Source: Int J Mol Sci. 2023 Sep 9;24(18):13904. doi: 10.3390/ijms241813904 (PMC10530982; doi:10.3390/ijms241813904)
Supplement: Supplementary file 1 [file ijms-24-13904-s001.zip › Supplementary Figures.pdf]

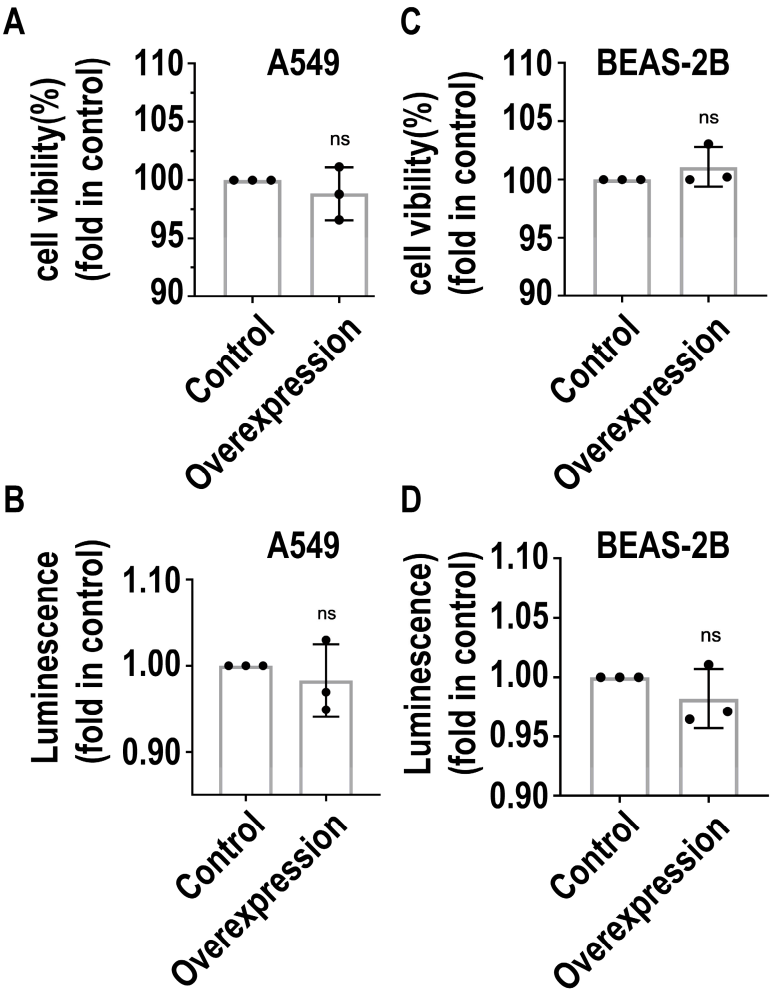

Figure S1. The effect of LncRNA AL137782.1 on the proliferation of alveolar epithelial cells. (A-B) CCK-8 assay was used to detect the effect of AL137782.1 on the proliferation of A549 and BEAS-2B cells; (C-D) CellTiter-Lumi™ luminescence assay was used to detect the effect of AL137782.1 on the proliferation of A549 and BEAS-2B cells. “ns” means no significant difference.
