# Supplementary material for: Novel Long Non-Coding RNA (lncRNA) Transcript AL137782.1 Promotes the Migration of Normal Lung Epithelial Cells through Positively Regulating LMO7
Source: Int J Mol Sci. 2023 Sep 9;24(18):13904. doi: 10.3390/ijms241813904 (PMC10530982; doi:10.3390/ijms241813904)
Supplement: Supplementary file 1 [file ijms-24-13904-s001.zip › Supplementary Tables.pdf]

Table S1 Exons and introns of AL137782.1

Number	Exon / Intron	Start	End	Length
	5′upstream sequence			
1	ENSE00002616327	75,549,773	75,549,862	90
	Intron 1-2	75,549,863	75,549,975	113
2	ENSE00003533156	75,549,976	75,549,987	12
	Intron 2-3	75,549,988	75,560,752	10,765
3	ENSE00003484188	75,560,753	75,560,881	129
	Intron 3-4	75,560,882	75,566,694	5,813
4	ENSE00003549073	75,566,695	75,566,851	157
	Intron 4-5	75,566,852	75,567,226	375
5	ENSE00003610299	75,567,227	75,567,312	86
	Intron 5-6	75,567,313	75,569,459	2,147
6	ENSE00003646379	75,569,460	75,569,507	48
	Intron 6-7	75,569,508	75,594,914	25,407
7	ENSE00003685022	75,594,915	75,594,990	76
	Intron 7-8	75,594,991	75,621,762	26,772
8	ENSE00002579899	75,621,763	75,621,868	106
	Intron 8-9	75,621,869	75,713,181	91,313
9	ENSE00003514330	75,713,182	75,713,252	71
	Intron 9-10	75,713,253	75,727,028	13,776
10	ENSE00003523650	75,727,029	75,727,098	70
	Intron 10-11	75,727,099	75,760,931	33,833
11	ENSE00003521160	75,760,932	75,761,038	107
	Intron 11-12	75,761,039	75,795,400	34,362
12	ENSE00003599174	75,795,401	75,795,431	31
	Intron 12-13	75,795,432	75,796,635	1,204
13	ENSE00003460049	75,796,636	75,796,749	114
	Intron 13-14	75,796,750	75,800,683	3,934
14	ENSE00003570891	75,800,684	75,800,882	199
	Intron 14-15	75,800,883	75,804,288	3,406
15	ENSE00002586842	75,804,289	75,807,120	2,832
	3′downstream sequence			

Table S2 The ORFs of lncRNA AL137782.1.

**Label**	**Strand**	**start**	**stop**	**Length(nt/aa)**
ORF1	+	49	711	663 丨 220
ORF2	+	1354	1464	111 丨 36
ORF3	+	1486	1647	162 丨 53
ORF4	+	1774	1863	90 丨 29
ORF5	+	2194	2319	126 丨 41
ORF6	+	2446	2874	429 丨 142
ORF7	+	146	268	123 丨 40

ORF8	+	2513	2608	96 | 31
ORF9	+	2612	2707	96 | 31
ORF10	+	3053	3157	105 | 34
ORF11	+	3230	3382	153 | 50
ORF12	+	4037	>4126	90 | 29
ORF13	+	648	1556	909 | 302
ORF14	+	1635	1757	123 | 40
ORF15	+	1836	2258	423 | 140
ORF16	+	2316	2465	150 | 49
ORF17	+	3597	3809	213 | 70
ORF18	+	3828	3914	87 | 28
ORF19	-	2784	2545	240 | 79
ORF20	-	1542	1408	135 | 44
ORF21	-	1251	1051	201 | 66
ORF22	-	555	430	126 | 41
ORF23	-	3473	3384	90 | 29
ORF24	-	3344	3060	285 | 94
ORF25	-	1652	1545	108 | 35
ORF26	-	1202	1077	126 | 41
ORF27	-	1049	933	117 | 38
ORF28	-	911	750	162 | 53
ORF29	-	179	57	123 | 40
ORF30	-	2743	2642	102 | 33
ORF31	-	2422	2327	96 | 31
ORF32	-	2074	1949	126 | 41
ORF33	-	805	557	249 | 82

Table S3 The sequence of lncRNA AL137782.1 after RACE

**RNA name**	**RNA sequence (5′-3′)**
AL137782.1	ATCTAGATGCATTCGCGAGGTAGGATCCCGCCCGGGGGAATTCGTAGGCG<br>CCGGTCACAGCTTGGATCTAGACGGCGGACGCAGTTCAGGAGGCACCAC<br>AGGCGGGAGGCGGCAGAACGCGACTCAACCGGCGTGGATGGCGGCCTC<br>AGGTAGGGCGGCGGGCGCGTGAAGGAGAGATGCGAGCCCCTCGAAGCT<br>TCAGCTGTGTTCTGGCGGCAAACCCGTTGCGAAAAGCCCCATGTACTCTG<br>CGTTGATACCACTGCTTGCCCTATAGGATGAGACAGAGTCTCACTCTGTCG<br>CCCAGGCTGAGTGCAGTGGCGCCATCTCTGCTCACTGCAACCTCCGCCTC<br>CTGGGTTCAAGCAATTCATCTCCCTCAGCCTTCCGAGTAGCTGGGATTAC<br>AGGGGCCCCCTATCATGCCTGGCTAATTTTTGTATATTTAGTAGAGATAAG<br>GTTTCACCATTTTAGCCAAGCTGGTCTCAAACTCCTGACCTCAAGTGATCC<br>ACCTGCCCTGGCCTCCCAAAGTGCTGGGATTACAGGCGTGAGCCACCCC<br>GCCTGGCCCCTGGTATCCCTTTCTCATTCCATTTGCCACAGTGGCCTAAGT<br>GTCCCCTGAAGAAGAAGGAATAATCTTTGAAAGTTTCACAAGTTGTGCAT<br>CATGCACTTTATGCAATAGGTATATGTAATCAGCAGGAAAAAGGAAAAT

	AAATTGCTCTGAAGTTTTATAAAAGAGCTCAAGGAATCTTCCCTTTGCATT<br>TTCCCTAGACTTGCCCTTCCCAGTCTCCACCTGAAAGTTATTTCCTTTTTCC<br>ACATCTCCACACACAACAAAACCGTGAAATTAGGTAAACTTTCTAACAA<br>GCCTTTTTGAACCGGCTTTTCAAAAGAACTGCCACATGTAAATATATCCTG<br>TCTCACACATTATTGTGCTAAAGAAGAATGTTGAATCCTACAGACTTCCA<br>AGCTGATGTAGTTGCACAGAAAGCAGCGTGTTATACATTACGCTTTTGTAC<br>TAAGTCCACTAAGTGAGTCAGGTTTCCTGATTCAGTGCAATCAGGTCCCA<br>GGCTAATGGAGCAGCAGAAGCAGGCAGAGGGTTGTCTGAGGCCAGACC<br>CGCAGCAGGCAGGCTCATGCAGACAGGGGCACCTGGGCTCATCCGAAGC<br>ACTACTGCTTCTCCTGGCTGCATGGGAGTCCGGTGATTTTACAGCCCGTTT<br>GGTAGCTAATTCCAGCAAGAGTGAAGGTCTTTGGCAGTTGCCGTCTAGCC<br>TATGCTCTGCTTTTTGTGCTCTACTAAAAACGGTGGCGCTTCTGTCAGAAC<br>CTGAGTCCTCATCCTCAGAGTCTGAATTCCTTCTACTGTGAGTGACATGAA<br>GAAAAGAAGGGAAGAGGGAATGAGACAAAGAATTGGAGAAAAACAGA<br>ACTACTAAAAATAATGCCGTGCTATGACTATGTATAGAACAGGGCTTTTTA<br>AGATCTAGATCCTCAGGTTTAAATTTAAACAGAAATTCATTTCCATTATATA<br>GGTTCCGCAGATACTAGAGAACTAATTAGACTTTCTCTGTCTCTCTTATTAT<br>TATAAAAAACATGCACATAGAACCCCAGCTGGGGTACTGAATGAACACT<br>GGTGTGACAGAAACATGCACAACGACAAACCTGAATCCCTGAAATTCTT<br>TATACCATGGCTTATAAGTTTCCCTTTTTATTCTTTTCCAGTTCACATCTTCT<br>GGGGTCCAACATTTGGGAAGAAACTGATCAAAAGCATTCCCTGGGTTTG<br>GCATGACTGGACTGAGCTTGCGCACATAAAGATCATCCTTTACAATGTTG<br>GGTATGCTTCTTGTCTTTGCTTTTTCCTCTTCCAAATAACAAGAGGACTTTG<br>AAGTTCCTTGTACAGTTGGCCAGTTCTCCACATTGGTGCCCCAAATCCTCC<br>TCTGATTACTGTTCAAAACATCCAACCGGTTAAGACACTGTATGAGCATTT<br>GTGACACAGAACACAAATGGCATGCTTTCTGGTTCACAAAGTTTACACCA<br>CTGTGAACAAAGCAAATTAGTTTCCATTCTCATCACCACCACAGCTCCTA<br>GGACAAAGTTATTATAACAGCATCTCATTTTAACTCAAGGATAGCTAGATA<br>CACACGGGCCAAAGATACTTCATTAGAAGCTGTGTTAAAAGGAAGAGGC<br>ACATAACAAACAATTTGGGGTGTTACTTTCTGAATTAAAATCAGCAGCAT<br>AATAGACAGCCAGATCCAGGACAAGTATTTCTGACCTCGTCAGTCCATAT<br>AGCCCCATGGCAAACATATCAGCAGGAGAAAATATTTGCGGACAAGAAT<br>TAGGATAGGTGATTTTCGAGATGAAATTCTGGGGCCTCTTTTTCACTTGGA<br>ATCTACGGTTGGCTAAGTCATCCAAAACTATGTCAGGAAACCTTCGTTCAT<br>CCTCAGAGTCCGAACCACTTTCAAAACCGTACGCCTCCCCATGAGAGGA<br>AGCTGGATTTGCAAAACCACACTCTGTTCTGCTAATTTGCTGAGAAATTA<br>AAGGATTCTTTTCATGTCCACTAAGATCAATCAAAAAAGGAAAAACATTA<br>CATGGCATGGCTCATCATGAACAATACAAATTATCTGGTAAGCTGAAAAA<br>TACCAATAAACTGAAGTCATGCATCTAAAAAAAGACCTAGCAAATCATAT<br>GCTGACATTCTAAAACAGAAGAAATGGCTTCAAGATATGTATAACTAAAA<br>ATAGAGGGATGAAAAATATTCTCACTAGTCAAGCTTAATATATGATCTATG<br>AAACACATTTAAGAGCCATTCTTTTTACCACATTTAACATCCTACCATATTC<br>GAAAAGCATACGAAACTCAATAATCACACCCATCTCAGTACCTTGAAAAT<br>GTTCCATCTGCTTCTGTATAAACCGGGCTTGCCCAACTTCTTCTGTTATCCT

	CATGTTTGTCTGGCTTTTTCTTTCTCAGAGGTGCTGGTACATAGGATGGCTG
	TCTACTTTTGTTGGGTAAAAAACGATTGAAGGGTAACGCAGTCTTTGGCT
	CAACAGCCGAAATCCTTCGATACGACATATCATCTTTATTATAATCCTGCAT
	CTTAAATGTAAATTCCGAATCTGTGTCACTTTCAAAACCTTCACGCCCCCC
	TCTCAACGTGATATCAGAGGAGCAGCTTGTCAATGACCTCGAGCCCAAA
	GAGTCCAAGCTTTCAAAGGAATCTTCTCTCTTATGGTGCCTTGGAGGAGC
	AAGAAATTCTCCACGTTCAGGACACCAGATGTCACCGTAGCCACTGTCCC
	TGCCACTTCTTTTCAGGAAGCTGGAGTCTTCGAGTGCCTTCGTCAGTGCTT
	GTCCTAAAAGATTCTCAAACGCTTTCAAATTAAGATGGGGACCATTATAG
	TACGGGTTGCTTTGTGCTTTTCTTCCCAGCCAGTACAATGTTATCAAAACA
	TTTTTCACTCTCCTGTCAGTCTCTTCTTGCTTGACAGTGACTCGATTTGATA
	AATCCTGTAGATCTCCAGGATGGAAAAGCTGGGCTTCTTTCAATCCAATCT
	GTTCACAAGCTTTCAAGAAAACGTTTATATTATCCAATCCTGCTATTGGTG
	TAGACAGTCTATTGATCTTCTTAATGACGCCAGGTTTAAGCTTATTAATCA
	AATCACACAGCAGAACACCATTTTCTAGAGAGGCTCGAAAATCTTTTGTT
	TCAAAATTCTTCTCTGTTACTGCCTTTTTTGCTGCAGACTCTGTCCCTGAGA
	TACAGCCAACCCACACAGATGCAGACATGAGCACATATCAGTTGCCTCC
	AAATTTCCAGAGCTCCGAGCTCTGTTCTCTAATTCATAGAGATGCCCATCT
	ACATGAACTAATGCAATAAAATGAAGATCTACTTTCTCATCTATACTTGGT
	GCCTCAGTCTGACCTTCATGGGCACTGGTCTCATGAGTAACTCGGATGGC
	ATCATAGTTCTCCAGGTATCTGGCTCGTTCTTCAGGGCTCATTGACACAGA
	TTCCTCCAGGAATTTTTTCAAGGTTGATCCAGATTCAAAGTGCATCTTGTC
	TTTATTGTTTGCAATAGCATGAATCAGTCCAATTGTTCCACAGGCATTGCT
	GATTGTTTGCTTCATGAAATATACTGATGATGTAACATCTTGTCCCTGAGAT
	TTTATTTTTTCTTCCTCTTCTGTTCTGAATACTTCATACTTTTCTGTAATAGG
	AAAGAGAAGTAAGACTGCACAGACTGGTCTTGGTACCATGCTAAGGAGT
	TCAGGATCCATTCCATATACATCAACGAATTGCCAGTTAGGATGTAGACCT
	AATTGTTTAAGAAACTGGTTGGTGACCTCGGGATTGGCCTCCAGCGGCAG
	CCAGCGTTGACCCTCCATGGCCGCGGTGCCCGGCCCTCCAGCTCTGACAG
	CCGCCGCCTTCGCCGC

Table S4 LncLocator prediction result

Subcellular locations	Score
Cytoplasm	0.496916218173
Nucleus	0.475012952868
Ribosome	0.005572040796
Cytosol	0.013560497258
Exosome	0.008938286943
